# Measuring Economic Freedom: Better Without Size of Government

**DOI:** 10.1007/s11205-016-1508-x

**Published:** 2016-11-30

**Authors:** Jan Ott

**Affiliations:** Erasmus University Rotterdam, Rotterdam, The Netherlands

**Keywords:** Big government, Convergent validity, Economic freedom, Fraser Institute, Freedom, Freedom House, Gallup World Poll, Global freedom, Happiness, Heritage Foundation, Predictive validity, Personal autonomy, Quality of government, Size of Government

## Abstract

The Heritage Foundation and the Fraser Institute measure economic freedom in nations using indices with ten and five indicators respectively. Eight of the Heritage indicators and four of the Fraser-indicators are about specific types of institutional quality, like rule of law, the protection of property, and the provision of sound money. More of these is considered to denote more economic freedom. Both indices also involve indicators of ‘big government’, or levels of government activities. More of that is seen to denote less economic freedom. Yet, levels of government spending, consumption, and transfers and subsidies appear to correlate positively with the other indicators related to institutional quality, while this correlation is close to zero for the level of taxation as a percentage of GDP. Using government spending, consumption transfers and subsidies as positive indicators is no alternative, because these levels stand for very different government activities, liberal or less liberal. This means that levels of government activities can better be left out as negative or positive indicators. Thus shortened variants of the indices create a better convergent validity in the measurement of economic freedom, and create higher correlations between economic freedom and alternative types of freedom, and between economic freedom and happiness. The higher correlations indicate a better predictive validity, since they are predictable in view of the findings of previous research and theoretical considerations about the relations between types of freedom, and between freedom and happiness.

## Introduction

The role of governments, in relation to security and freedom, has been a subject of vivid discussions since Thomas Hobbes published his ‘Leviathan’ in 1651. Utilitarians like Jeremy Bentham, James Mill and John Stuart Mill added happiness as an additional value to be considered. We cannot decide, in any scientific way, what priorities security, freedom, and happiness deserve as values, but we can try to get a better understanding of their mutual relations as actual phenomena.

### Previous Research by Veenhoven

In his article ‘Freedom and Happiness; a comparison of 126 nations in 2006’ with data for the years 2000–2006 Veenhoven (2008) defines freedom as the possibility to choose. This possibility requires an opportunity and a capability to choose. The capability is an individual characteristic, but the opportunity depends on the environment.^1^

This opportunity, offered by the environment, involves two requirements: first that there is something to choose. This requirement depends on the societal supply of life style alternatives and conditions like information and physical infrastructure. The second requirement is that free choice is not frustrated by formal or informal restrictions created by other people or institutions. It is not unusual to discern positive and negative freedom, parallel to this distinction. Positive freedom refers to the actual availability of options; negative freedom refers to the absence of restrictions or interference by others.^2^

The focus of Veenhoven is on negative freedom in nations by the absence of formal or informal restrictions. He discerns three kinds: economic freedom as measured by the Heritage Foundation, global freedom^3^ as measured by Freedom House, and private freedom. Veenhoven measures private freedom with an index for the absence of restrictions to travel, religion, marriage, divorce, euthanasia, suicide, homosexuality, and prostitution. He uses data of the World Values Surveys to apply this index.

One of Veenhoven’s conclusions is that, in nations, these types of freedom tend to go together. There is a substantial mutual correlation: +.69 between economic freedom and global freedom; +.66 between global and private freedom, and +.58 between economic and private freedom. Another conclusion is that there is a positive correlation between freedom and average happiness; for economic freedom +.63;^4^ for private freedom +.58; and for global freedom +.54. Together these kinds of freedom explain 44% of the variation in average happiness in nations. Veenhoven observes that the positive correlation is universal, even though the correlation is somewhat higher for rich nations and nations with higher levels of education.

This correlation is in his view at least partly the result of causal effects of freedom on happiness. He suggests that global freedom, or political freedom in his terminology, contributes to economic freedom, and economic freedom contributes to happiness. Both types of freedom make way for private freedom, which on its turn adds to happiness by allowing a better fit between life-styles and individual preferences. This conclusion is consistent with more findings that freedom and individual autonomy are important for happiness and development (Sen 1999). There are no signs that the impact of freedom on happiness is lower at higher levels of freedom; so there are, so far, no signs of ‘diminishing returns’ (Veenhoven 2008).^5^

## Research Questions

In this research-paper I want to repeat Veenhoven’s research with data for the years 2010–2012, but I want to answer some additional questions. Veenhoven did not assess how economic freedom is measured. The Heritage Foundation and the Fraser Institute assume that government activities have, on the balance, a negative impact on economic freedom. These institutes use general levels of government activities, as reflected in expenditures, consumption and taxation, as negative indicators. This assumption is debatable. I will therefore answer the next research questions.

1. Do we get similar results as Veenhoven for 2000–2006 if we use data for 2010–2012?
2. Are there options to improve the measurements of economic freedom by the Heritage Foundation and the Fraser Institute?
3. What is the impact of such improvements on the correlation between economic freedom and alternative types of freedom, and on the correlation between economic freedom and happiness? In view of previous research, and the related theoretical considerations, we may expect higher correlations if economic freedom is measured in a better way.

To answer the research-questions I use a sample of 127 nations. Nations with missing values for more than one key-variable are left out. There are almost 200 countries in the world and these 127 countries are not a random sample, because there are more missing values among ‘failed states’ without any effective government. This has, however, no substantial negative impact on the representativeness of the sample.^6^ The representativeness of the 127 countries is good enough to justify general conclusions. Average scores are used for the years 2010–2012 (around 375 country-year observations).

In the Appendices 1, 2, 3 and 4 some information about relevant variables and data is summarized. More information is available at the sites mentioned. Descriptive statistics and correlations are presented in Appendices 5 and 6. The scores for global freedom and press-freedom (Freedom House) are reversed to make sure that higher scores always indicate more freedom. This makes it easier to understand the correlations in the Tables.

The research questions 1, 2 and 3 will be discussed in Sect. 3, 4 and 5; followed by conclusions and a discussion in Sect. 6.

## Comparing results for 2000–2006 and 2010–2012; types of freedom, and types of freedom and average happiness, go together

To answer the first research question we compare the correlation between different types of freedom, and between these freedoms and happiness, for 2000–2006 and for 2010–2012. We compare private freedom for 2000–2006 with personal autonomy for 2010–2012. This is acceptable considering the overlap in indicators (see Appendix 2, section Global Freedom and Personal Autonomy). The results for 2010–2012 in Table 1 are in brackets.

**Table 1:** Correlations in 2000–2006 and in 2010–2012; correlations in 2010–2012 in brackets

	Economic freedom (Heritage Foundation)	Index private freedom (personal autonomy)	Global freedom^a^ (political rights and civil liberties)
Economic freedom (Heritage Foundation)	X		
Index private freedom (personal autonomy)	.58 (.65)	X	
Global freedom^a^ (political rights and civil liberties)	.69 (.58)	.66 (.92)	X
Happiness	.63 (.54)	.58 (.60)	.54 (.53)

120–127 nations

All correlations are positive and significant at .01 level

^a^Scores reversed; higher scores always indicate more freedom

In the cells of Table 1 we see similar correlations for 2000–2006 and 2010–2012. The correlation between global freedom and personal autonomy (+.92) is higher than between global freedom and private freedom (+.66). This is understandable since personal autonomy is a sub-indicator of civil liberties, one of the two components of global freedom. The correlations are otherwise comparable and we may conclude, following Veenhoven, that different types of freedom go together and go together with more happiness. We will see the same pattern with more types of freedom in Table 5a, b.

The empirical observation that different types of freedom go together, and go together with more happiness, deserves some attention because this is not ‘self-evident’. Specific types of freedom can be more important for specific groups. More negative freedom will be more important for people with money and power (no interference please!); more positive freedom will be more important for poor people (more public goods and services please!); more economic freedom will be more important for employers, the self-employed and investors (just rule of law, no regulations please!); more private freedom and personal autonomy will be more important for cultural, religious, and sexual minorities (more equality and tolerance please!).

Such differences in the appreciation of freedom are apparently not inconsistent with a high mutual correlation and a high correlation with average happiness. One reason is perhaps that there is a substantial overlap in (sub-) indicators in the measurement of different freedoms. There is also a more theoretical explanation: it is eventually always about the individual freedom to make decisions, and we may expect that individuals will claim similar levels of freedom, whatever the decisions at stake. A certain level of individual freedom or autonomy can easily become a general cultural standard.

## The Measurement of Economic Freedom and Options for Improvement

### Using Convergent Validity to Evaluate Measurements

I use convergent validity to evaluate measurements. This validity refers to the consistency of a measurement, if a variable is measured by several indicators. The Cronbach alpha, as a scale reliability coefficient, is a good statistical measure for this consistency.^7^ The minimum value of this measure is 0 and the maximum value is 1. The value will increase when correlations between the indicators increase. As a general rule a value of 0.7 is acceptable and a value of 0.8 is good.

### Measurement by the Heritage Foundation (Table 2)

Column 1, 2 and 3 in Table 2 are about the convergent validity of the measurement of economic freedom by the Heritage Foundation. In the first column are the correlations between the aggregated or summary scores for economic freedom in nations, as presented by the Heritage Foundation in the original index, and the scores of nations on the 10 indicators, mentioned in the beginning of the rows, used by this Foundation to construct these summary scores. Eight indicators are related to specific types of institutional quality; two indicators (3 and 4) are related to levels of government activities. Nations with higher levels of institutional quality or higher levels of government activities always get higher scores for these indicators.

**Table 2:** Heritage Foundation: convergent validity

	Correlations with Original Index, with Index without 3 and 4, and with Index with 3 and 4 reversed, in column 1, 2 and 3.		
	Original Index (1)	Index, without 3 and 4 (2)	Index, 3 and 4 reversed (3)
Original Index	X		
Index, without 3 and 4	+.96	X	
Index, 3 and 4 reversed	+.87	+.97	X
1. Property rights	+.85	+.92	+.93
2. Freedom of corruption	+.81	+.90	+.92
3. Fiscal freedom	−.00 (ns)	X	+.42
4. Government spending	+.11 (ns)	X	+.62
5. Business freedom	+.78	+.82	+.80
6. Labor freedom	+.52	+.48	+.39
7. Monetary freedom	+.62	+.64	+.61
8. Trade freedom	+.77	+.78	+.73
9. Investment freedom	+.81	+.84	+.82
10. Financial freedom	+.84	+.87	+.83
Cronbach alpha	.75	.90	.88

125–127 nations

All correlations significant at .01 level, only the correlations of Fiscal Freedom (3, level of taxation as %GDP) and government spending (4) with the summary scores in the Original Index are not significant (−.00 and +.11)

In the first column (1) there are correlations between a and b

(a) The scores for actual levels of 10 indicators mentioned in the rows. Nations get higher scores if they have higher levels of taxation and government spending (3 and 4) and higher scores for the institutional qualities (1, 2 and 5, 6, 7, 8, 9, 10)

(b) The aggregated or summary scores for economic freedom in nations as presented by the Heritage Foundation (Original Index). These scores are the average of the scores for 10 indicators mentioned in the rows. Nations with higher levels of taxation or government spending get lower scores for indicators 3 and 4 and, as a consequence, lower scores for economic freedom. Nations with higher levels of institutional qualities get higher scores for the indicators 1, 2, 5, 6, 7, 8, 9 and 10

In the second column (2) there are correlations between

(a) Same as in column (1), but not for 3 and 4

(b) Same as in column (1) but now the scores are the average of 8 indicator 1, 2 and 5, 6, 7, 8, 9, 10. Scores for 3 and 4 are left out

In the third column (3) there are correlations between

(a) Same as in column (1), 3 and 4 included

(b) Same as in column (1) but now the scores are the average of the 10 indicators again mentioned in the rows, but now nations get higher scores for higher levels of taxation and government spending, and as a consequence higher scores for economic freedom. Nations with higher levels of institutional qualities (1, 2 and 5, 6, 7, 8, 9, 10) get again higher scores if they have higher levels of institutional qualities

There are, however, different ways to construct the summary scores. In the first column we look at the construction by the Hermitage Foundation. The summary scores are the average of all indicators with equal weights. The levels of government activities, however, are used as negative indicators for economic freedom; nations get lower scores if these levels are higher. If the assumption of the Heritage Foundation, that government activities have a negative impact on economic freedom, is correct, we may expect substantial negative correlations between these actual levels of government activities and the summary scores. In the first column we see however that the actual correlation is indeed negative, but close to zero, for ‘Fiscal Freedom’ or the level of taxation (%GDP), and positive for ‘Government Spending’ (%GDP). Both correlations are not significant while all other correlations are positive and significant (at .01 level). The validity of this measurement with 10 indicators is nevertheless acceptable; the Cronbach alpha is .75. There are two options for improvement.

### Options for Improvement

- Leaving indicators 3 and 4 out. In the second column indicators 3 and 4 are left out in the construction of the summary scores. The summary scores are now the averages of the 8 remaining indicators related to institutional qualities. We see a substantial improvement in the convergent validity. The Alpha goes up from .75 to .90.
- Reversing the sign of 3 and 4. In the third column we reverse the scores for the indicators 3 and 4 in the construction of the summary scores. We have again 10 correlations between summary scores and 10 indicators, but now nations also get higher summary scores if they have higher levels of government activities, instead of lower scores. Indicators 3 and 4 always remain the same: higher scores are related to higher levels of actual government activities. We see a similar improvement in the convergent validity. The correlation between the summary scores and actual levels of taxation and government spending is now positive and significant. The Alpha goes up from .75 to .88.

We may conclude that both options improve the validity.^8^ It is clear that the assumption that actual levels of taxation and government spending have a negative impact on economic freedom, as measured by the other indicators, is not correct. The correlation between level of taxation and such qualities is negative but close to zero. The correlation between government spending (expenditures), with government consumption and transfers and subsidies as important components, is positive but not significant. It is however debatable to use taxation and government spending as positive indicators for economic freedom. There is no theoretical justification; government spending, consumption and transfers and subsidies included, can be directed at very different policies, liberal or less liberal!

### Measurement by the Fraser Institute (Tables 3, 4)

Columns 1, 2 and 3 in Table 3 are about the convergent validity of the measurement of economic freedom by the Fraser Institute. In the first column are the correlations between the summary scores for economic freedom in nations, as presented by the Fraser Institute (Original index), and the scores of nations on the 5 indicators of this Institute to construct these summary scores. Four indicators are related to specific types of institutional quality; one indicator is related to actual levels of government activities (‘Size of Government’). Nations with higher levels of institutional quality and higher levels for ‘Size of Government’ always get higher scores for these indicators.

**Table 3:** Fraser Institute: convergent validity

	Correlation with Original Index, with the Index without Size, and with the Index with Size reversed, in column 1, 2 and 3		
	Original Index	Index, without Size	Index, Size reversed
Original Index	X		
Index, without Size	+.95	X	
Index, Size reversed	+.81	+.96	X
1. Size of Government^a^	−.17 (ns)	X	+.44
2. Legal system and property protection	+.75	+.86	+.89
3. Sound money	+.82	+.84	+.79
4. International trade	+.88	+.90	+.82
5. Regulation	+.76	+.76	+.69
Cronbach alpha	.66	.85	.76

125–127 nations

All correlations significant at .01 level; except for the correlation between Size of Government and the scores in the Original Index (−.17, not significant)

^a^Actual aggregate level of (a) government consumption as a % of national consumption, (b) transfers and subsidies as a % of GDP, (c) government enterprises and investment as a % of GDP, and (d) top tax-rates (average, equal weights, see Table 4)

In the first column (1) there are correlations between a and b

(a) The scores for actual levels of 5 indicators mentioned in the rows. Nations get higher scores for Size of Government (indicator 1) if they have higher levels of government consumption, transfers and subsidies, government enterprises and top tax-rate. Nations get higher scores for indicators 2, 3, 4, 5 if they have higher levels of these institutional qualities

(b) The aggregated or summary scores for economic freedom in nations as presented by the Fraser Institute (Original Index). These scores are the average of the scores for 5 indicators mentioned in the rows. Nations with higher scores for Size of Government get lower scores for indicator 1, and, as a consequence, lower scores for economic freedom. Nations with higher levels of institutional qualities get higher scores for the indicators 2, 3, 4, 5, and as a consequence, higher scores for economic freedom

In the second column (2) there are correlations between

(a) Same as in column (1), but not for 1

(b) Same as in column (1) but now the scores are the average of 4 indicators (2, 3, 4, 5). Scores for Size of Government (indicator 1) are left out

In the third column (3) there are correlations between

(a) Same as in column (1)

(b) Same as in column (1) but now the scores are the average of the 5 indicators again mentioned in the rows, but now nations get higher scores for higher levels of government consumption, transfers and subsidies, government enterprises, and top tax-rate, and as a consequence higher scores for economic freedom. Nations with higher levels of institutional qualities (2, 3, 4, 5) get again higher scores if they have higher levels of institutional qualities

**Table 4:** Specification of Size of Government (Fraser Institute)

	Correlation with Original Fraser Index	Correlation with happiness
Size of Government* (average a, b, c, d)	−.17*	+.28**
a. Government Consumption^a^	+.18*	+.50**
b. Transfers & Subsidies as a %GDP	+.29**	+.49**
c. Government Enterprises & Investment as %GDP	−.52**	−.41**
d. Top Tax rate^b^	−.20*	+.14 (ns)

125–127 nations

* significant at .05%

** significant at .01%

^a^Actual level of government consumption as a % of national consumption

^b^Average of top marginal income tax rate and the top marginal income and payroll tax rate, and the income threshold at which these rates begin to apply. The measurement of the Heritage Foundation is more comprehensive, because it refers to the total tax burden as a % of GDP

There are, however, different ways to construct the summary scores. In the first column we look at the construction by the Fraser Institute. The summary scores are the average of all 5 indicators with equal weights. ‘Size of Government’ however, is used as a negative indicator for economic freedom; nations get lower scores if they have more government. If the assumption of the Fraser Institute is correct, that government activities have a negative impact on economic freedom, we may expect a substantial negative correlation between actual ‘Size of Government’ and the summary scores.

In the first column we see indeed that the correlation is negative but not significant. The negativity is consistent with the assumption of the Fraser Institute that ‘Big Government’ has a negative impact on economic freedom. The negativity is strange, however, because ‘Size of Government’ contains ‘Government Consumption’ and ‘Transfers and Subsidies’ as important components. Taken together these two sub-indicators are comparable with the actual level of government spending as used by the Heritage Foundation. This actual level of spending has a positive correlation with the summary scores for economic freedom as measured by the Heritage Foundation.

In Table 4, about the sub-indicators of ‘Size’, we see the explanation. Actual levels of government consumption and transfers and subsidies have indeed a positive correlation with the summary scores of the Fraser Index. This ‘positivity’ is overruled, however, by ‘Government enterprises and Investment’ (%GDP) and ‘Top Tax-rate’ (average, equal weights). The last two indicators have substantial negative correlations with the summary scores of the Fraser Index.

The validity of the measurement in the first column in Table 3 is reasonable; the Cronbach alpha is .66. But there are options for improvement.

### Options for Improvement

- Leaving indicator 1 out. In the second column indicator ‘Size’ is left out in the construction of the summary scores. The summary scores in the Index are now the averages of the 4 remaining indicators related to institutional quality. The Alpha goes up from .66 to .85, indicating a better convergent validity.
- Reversing the sign of indicator 1, ‘Size of Government’. In the third column we reverse the scores for ‘Size of Government’ in the construction of the summary-scores. We have again 5 correlations between summary scores and 5 indicators, but now nations get higher summary scores if they have higher levels of actual government activities, as expressed in a higher score for ‘Size of Government’. The correlation between the summary scores and scores for ‘Size’ is now positive and significant (at .01 level), and the Alpha goes up, but not as much as in option a, from .66 to .76.

Both options improve the convergent validity, but the second option, reversing the sign of ‘Size’ is less effective, obviously because of its mixed composition as shown in Table 4. We may conclude that ‘Size’ is not an appropriate indicator to measure economic freedom. Only actual level of ‘Government Enterprises’ (%GDP) and ‘Top Tax-rate’ can be used as negative indicators, because they have a significant negative correlation with economic freedom, as measured by the other indicators. Both indicators are however rather specific, and not representative for government activities in general. ‘Top Tax-rate’ is rather specific because it is technical characteristic of a tax-system. The level of taxation, as measured by the Heritage Foundation, is more comprehensive because it refers to the total tax burden as a % of GDP. The negative correlation between this tax burden, as measured by the Heritage Foundation, and economic freedom is, however, close to zero and not significant.

The inconsistency of ‘Size of Government’ is also visible in the correlation of the sub-indicators with happiness. This correlation is positive for government consumption, transfers and subsidies^9^ and taxation, but negative for government enterprises and investments.^10^

## The Impact of a Better Measurement of Economic Freedom on the Correlation with Alternative Freedoms and Happiness

Reversing indicators related to levels of government activities, and using them as positive instead of negative indicators, is not a good option. I therefore only assess the impact of leaving them out since this option is more effective and transparent.

In Table 5a, b, for the Heritage Foundation and the Fraser Institute respectively, we see that leaving levels of government activities out as indicators in measuring economic freedom leads indeed to higher correlations with alternative types of freedom and happiness. The higher correlations indicate a better predictive validity, since they are consistent with findings of previous research and theoretical considerations.

**Table 5:** Correlations of economic freedom (a) (Heritage Foundation) with alternative freedoms, satisfaction with freedom, and happiness, (b) (Fraser Institute) with alternative freedoms, satisfaction with freedom, and happiness

	Original Index	Index, Without 3&4	Global Freedom^a^	Personal Autonomy	Press Freedom^a^	Freedom Satisfaction
(a)						
Original Index	X					
Index, without 3&4	.96	X				
Global Freedom^a^	.58	.68	X			
Personal Autonomy	.65	.76	.92	X		
Press Freedom^a^	.58	.69	.93	.87	X	
Freedom Satisfaction	.45	.45	.33	.38	.34	X
Happiness	.54	.61	.53	.60	.50	.58
(b)						
Original Index	X					
Index, without size	.95	X				
Global Freedom^a^	.51	.59	X			
Personal autonomy	.57	.66	.92	X		
Press Freedom^a^	.50	.59	.93	.87	X	
Freedom satisfaction	.41	.45	.33	.38	.34	X
Happiness	.46	.55	.53	.60	.50	.58

120–127 nations

All correlations are positive and significant at .01 level

^a^Original scores reversed; higher scores always indicate more freedom or more satisfaction with freedom

Only ‘Freedom satisfaction’ (satisfaction with freedom to make life choices) has a relatively low correlation with the different types of actual freedom. The correlation between this satisfaction and economic freedom is also rather insensitive for the improvements in the measurement of economic freedom. This variable is, however, not really about actual freedom but about satisfaction with freedom. It is a subjective reality and as such it has a substantial correlation with (subjective) happiness. High correlations between individual subjective realities are quite common, but it is interesting to see a high correlation between average subjective realities in nations.

## Conclusions and Discussion

### Conclusions

Now we can answer the research questions, about the mutual correlations of types of freedom, possibilities to improve the measurement of economic freedom by the Heritage Foundation and the Fraser Institute, and the impact of such improvements on the correlation between economic freedom and alternative freedoms and between economic freedom and happiness.

1. We get similar results as Veenhoven who used 2000–2006, if we use data for 2010–2012; different types of freedom, economic freedom included, go together and go together with average happiness.
2. The measurement of economic freedom by the Heritage Foundation and the Fraser Institute is acceptable, but can be improved by leaving out levels of government activities. The only acceptable levels of government activities that can be used as negative indicators are the level of government enterprises and investments, as a % of GDP, and ‘Top tax-rate’ as measured by the Fraser Institute. These indicator are, however, rather specific and not representative for government activities in general. Taxation, as measured by the Heritage Foundation as a % of GDP, is more representative, but the correlation with economic freedom, as measured with institutional indicators, is close to zero. Levels of government spending, transfers and subsidies and government consumption, are also representative for government activities in general, but have a positive correlation with economic freedom as measured by the institutional indicators. They should certainly not be used as negative indicators. There is, however, no substantial theoretical justification to use them as positive indicators instead. As a general rule it is apparently better to use types of institutional quality to measure economic freedom, and to ignore the Size of Governments.
3. If economic freedom is measured in a better way we see, as expected, higher correlations between economic freedom and alternative types of freedom and between economic freedom and happiness.

### Discussion: the Importance of the Quality of Governments

In previous research I found that the correlation between government activities and happiness depends heavily on the quality of governments, and in particular on the technical or delivery quality. The conclusions in this research indicate that, in the sample of about 127 nations, this quality is ‘good enough’ to create a positive correlation, or at least a neutral correlation, between general levels of government activities on the one hand and economic freedom and average happiness on the other. Since this sample is representative for all 200 current nations we may assume that nowadays government activities in general contribute to freedom and happiness, even if there are some regrettable exceptions.

The quality of governments is a strong predictor of average happiness. Only GDP per capita has a comparable importance for happiness, but GDP and economic growth depend heavily on the quality of governments. Kaufman of the World Bank (2005) once estimated that a nation improving the quality of its governance from ‘low’ to ‘average’ can almost triple its income per capita in the long term. Up to a point GDP per capita is an intermediate between the quality of governments and average happiness. Such statements are obviously generalizations; there are exceptions. Bad governments can contribute to happiness if they have enough money. Governments with money can also improve their quality first and contribute to happiness later. We also must realize that small governments, with low levels of spending and transfers, can be very effective, e.g. if they can solve problems with intelligent legislation. Size and power are different subjects.

The quality of governments is important for happiness in two ways (Ott 2010). The quality is important in a direct way in direct contacts between citizens and government agencies: citizens want to be treated carefully and respectfully.^11^ The indirect impact depends on the creation of conditions and resources that contribute to happiness, like safety, physical infrastructure, employment, education and information. These conditions and resources also contribute to freedom. Freedom, economic freedom included, is apparently an important intermediate, just like GDP, between the quality of governments and happiness.

Some people are optimistic about the impact of governments on freedom and happiness. Other people are more pessimistic.^12^ Contacts between citizens are based on equality and consensus, but contacts between citizens and government agencies are based on hierarchy. Most of us prefer contacts based on consensus. And citizens can become inactive, and even apathetic, if their governments are too big or powerful.

It is absolutely wise to be critical about governments, but we have to be open minded. The observation that there is in general a positive or neutral relation between government activities and economic freedom, suggests that there is no natural contradiction between governments and free markets. Free markets need institutional qualities and some qualified and authoritative supervision. Government agencies can provide for such facilities. Free markets and governments are therefore not antagonistic, but need each other to produce the best outcomes in terms of freedom and happiness.

## Appendix 1: Economic Freedom as Defined and Measured by the Heritage Foundation and the Fraser Institute

The measurements of the Heritage Foundations and the Fraser Institute are very similar and primarily directed at negative freedom. Economic freedom is defined as the freedom of individuals to engage in economic transactions without interference. Most indicators value specific types of institutional quality, e.g. rule of law, the protection of property and sound money. A few indicators value ‘small government’ by using levels of government activities as negative indicators. Many data come from the same sources. The scores for countries are based on available statistics and on the standardized assessments of experts. It is no surprise that the correlation in the outcomes is high; +.88 for averages in the years 2010–2012. If the measurements are improved this correlation goes up to +.91.

In Measurement by the Heritage Foundation and Measurement by the Fraser Institute information is summarized about the measurement of economic freedom by the Heritage Foundation and the Fraser Institute.

### Measurement by the Heritage Foundation

#### Concept

Economic freedom is defined as the fundamental right of every human to control his or her own labor and property. Individuals are free to work, produce, consume, and invest in any way they please. Governments allow labor, capital, and goods to move freely, and refrain from coercion of liberty beyond the extent necessary to protect and maintain liberty itself.

#### Measurement

Economic freedom is measured with 10 indicators with equal weights, related to four broad categories.

A. Rule of Law:
  1. Property rights.
  2. Freedom from corruption.
B. Limited government:
  3. Fiscal Freedom (= total tax burden as % GDP).
  4. Government spending (% GDP).
C. Regulatory efficiency:
  5. Business freedom.
  6. Labor freedom.
  7. Monetary freedom.
D. Open markets:
  8. Trade freedom.
  9. Investment freedom.
  10. Financial freedom.

For each indicator countries can get 0–100 points and a summary score for economic freedom in general, as the average score of the 10 indicators. More points indicate more freedom. Countries get higher scores if they have lower levels of Fiscal Freedom (indicator 3, = level of taxation as %GDP) and government spending (indicator 4, %GDP). These levels are used as negative indicators.

#### Data Source

The Heritage Foundation collects information from many specific sources, like the World Bank, IMF and Economist Intelligence Unit. Data are available in the Index of Economic Freedom of the Heritage Foundation. See: http://www.heritage.org/index.

### Measurement by the Fraser Institute

#### Concept

Economic freedom implies that individuals are permitted to choose for themselves and engage in voluntary transactions, as long as they do not harm the person or property of others. The primary role of government is to protect individuals and their property from aggression. The index of economic freedom of the Fraser Institute is designed to measure the extent to which the institutions and policies correspond with a limited government ideal, where the government protects property rights and arranges for the provision of a limited set of ‘public goods’ such as national defence and access to money of sound value.

A country must provide secure protection of privately owned property, even-handed enforcement of contracts and a stable monetary environment. It also must keep taxes low, refrain from creating barriers to both domestic and international trade, and rely more fully on markets rather than government spending and regulation to allocate goods and resources. A country’s summary rating in the index is a measure of how closely its institutions and policies compare with the idealized structure implied by standard textbook analysis of microeconomics.

#### Measurement

Each year the Fraser Institute presents a report about economic freedom in nations: the annual ‘Economic Freedom of the World Report’ (EFWR). Economic freedom is measured in five major areas:

1. Size of Government, with four sub-indicators with equal weights: government consumption as a % of national consumption, transfers and subsidies, government enterprises and investments, and top tax-rate.
2. Legal structure and security of property rights.
3. Access to sound money.
4. Freedom to trade internationally.
5. Regulation of credit, labor, and business.

Within these five areas there are 23 components and many of them are made up of sub-components. Each component and sub-component is placed on a 0–10-scale. The sub-component ratings are averaged to determine each component and the component ratings are averaged to derive ratings for each major area. The final summary rating is the average of the five area ratings on a 0–10 scale; lower scores indicate lower levels of economic freedom. In area 1, Size of Government, countries get lower scores if they have higher levels of government consumption, transfers and subsidies, government enterprises and investments, and top tax-rates (=’bigger government’). These levels are used as negative indicators.

#### Data Source

The data-set of the EFWR-index is actualized each year with new data for the last year. The Fraser Institute collects information from more or less the same sources as the Heritage Foundation, e.g. the Doing Business dataset of the World Bank. See: http://www.freetheworld.com.

## Appendix 2: Global Freedom, Personal Autonomy, and Press Freedom, as Measured by Freedom House

The freedoms as measured by Freedom House are also primarily directed at negative freedom; the autonomy of individuals and the independence of the press without interference by the state or other external forces. The ratings are based on assessments by analysts using a broad range of sources of information, including foreign and domestic news reports, academic analyses, nongovernmental organizations, think tanks, individual professional contacts, and visits to the region. In Global Freedom and Personal Autonomy information is summarized about the measurement of global freedom and personal autonomy. Next section is about Press Freedom.

### Global Freedom and Personal Autonomy

#### Concept

Freedom House defines global freedom as the opportunity to act spontaneously in a variety of fields outside the control of the government and/or other centers of potential domination. It measures freedom according to two broad categories: political rights and civil liberties. The sum of the scores for political rights and civil liberties indicate the state of global freedom in nations as experienced by individuals.

Political rights enable people to participate freely in the political process through the right to vote, compete for public office and elect representatives who have a decisive impact on public policies and are accountable to the electorate. Civil liberties allow for the freedom of expression and belief, associational and organizational rights, rule of law, and personal autonomy without interference from the state. Personal autonomy is one of the indicators of civil liberties and is more specifically defined by the following aspects:

- Do citizens enjoy the freedom of travel or choice of residence, employment, or institution of higher education?
- Do citizens have the right to own property and establish private business? Is private business activity unduly influenced by government officials, the security forces, political parties/organizations, or organized crime?
- Are there personal social freedoms, including gender equality, choice of marriage partners, and size of family?
- Is there equality of opportunity and absence of economic exploitation?

This personal autonomy is comparable to Veenhoven’s ‘private freedom’; measured with an index for the absence of restrictions to travel, religion, marriage, divorce, euthanasia, suicide, homosexuality, and prostitution (using data of the World Values Surveys).

#### Measurement

Each country or territory is assigned two numerical ratings from 1 to 7 for political rights and civil liberties. Global freedom is the average of the two averages, also ranging for 1–7. Higher scores indicate less freedom. Countries with 1 or 2 points are ‘free’; with 3, 4 or 5 points ‘partly free’ and with 6 or 7 points ‘not free’. In the tables the scores are reversed to make them more consistent with the other scores. Higher scores for personal autonomy, on a scale ranging from 0 to16 points, indicate more autonomy.

#### Data Source

The Freedom of the world survey provides an annual evaluation of the state of global freedom as experienced by individuals. Legal rights are considered, but more emphasis is placed on whether these rights are implemented in practice. Rights and liberties can be affected by both state and non-state actors. In this analysis findings are used for the year 2010 and 2012 and are retrieved from the report ‘Freedom in the World 2015’. The data are stable over the years and there is always a high correlation between the scores for political rights and civil liberties; +.92 in 2012. See https://freedomhouse.org.

### Press Freedom

#### Concept, Measurement and Data

The Freedom of the Press report measures the level of media independence in 197 countries and territories. Each country receives a numerical score from 0 (the most free) to 100 (the least free) on the basis of combined scores from three subcategories:

A. The legal environment.
B. The political environment.
C. The economic environment.

For each category, a lower number of points is allotted for a more free situation, while a higher number of points is allotted for a less free environment. Here again this is reversed in the tables to make them more consistent and understandable. Data are available in: https://freedomhouse.org/report/freedom-press/freedom-press-2011#.VcN4j3kw_AU.

## Appendix 3: Happiness, and Freedom to Make Life Choices, as Measured by the Gallup World Poll

### Happiness

#### Concept

Following Veenhoven (2008) I define happiness as ‘the degree to which someone evaluates positively the overall quality of his or her present ‘life-as-a-whole’. In other words: *‘*how much one likes the life one lives*’.*

#### Measurement

Since happiness is something that an individual has in mind, it can be measured using questions. Many different questions are used; for an overview see the collection of Happiness Measures that is part of the World Database of Happiness (Veenhoven 2014). The present analysis draws on responses to a survey question, developed by Cantril (1965), which reads as follows: Suppose we say that the top of the ladder represents the best possible life for you and the bottom of the ladder represents the worst possible life for you. Where on this ladder do you feel you personally stand at the present time? Please use this card to help you with your answer.

**Table:**

0	1	2	3	4	5	6	7	8	9	10
Worst possible life								Best possible life		

The formulation best and worst possible life invites respondents to take into account all relevant domains of their life, like social relations, work, housing, leisure and so on. This question invites a comparative appraisal of life and measures the cognitive dimension of happiness in the first place.

Data available in the World Happiness Report; edited by Helliwell et al. (2015): http://worldhappiness.report/wp-content/uploads/sites/2/2015/04/WHR15_Sep15.pdf.

### Freedom to Make Life Choices, or ‘Satisfaction with Freedom’

Freedom to make life choices is not about actual freedom, like the previous types of freedom, but about the satisfaction with actual freedom. It is defined as the average of responses in a nation to the question: “Are you satisfied or dissatisfied with your freedom to choose what you do with your life?”

The formulation ‘Freedom to make life-choices’ is somewhat misleading. In the text and in the tables the formulation ‘Satisfaction with freedom’ or ‘Freedom Satisfaction’ is used. Higher scores indicate more satisfaction. Data available in the World Happiness Report 2015; see site in 3.1.

## Appendix 4: Quality of Governments as Measured by the World Bank

The World Bank evaluates every year six aspects of the quality of governments:

1. Voice and accountability
2. Political stability and absence of violence
3. Government effectiveness
4. Regulatory quality
5. Rule of law
6. Control of corruption.

It is possible to discern the democratic and the technical or delivery quality of governments. The democratic quality is the average score for the first two aspects and the technical or delivery quality is the average of the last four. (Helliwell and Huang 2008; Ott 2010). The correlation between the last four aspects is very high (> + .9). The correlation between the technical quality of governments and average happiness in nations is high and universal (Ott 2010).

Scores are standardized scores with an average of 0 and a standard-deviation of 1 in the original sample of the World Bank of almost 200 nations. Higher scores indicate a better quality. Data available in the World Happiness Report 2015; see site in 3.1. More information about the Worldwide Government Indicators is available at the World Bank site: http://info.worldbank.org/governance/wgi/index.aspx#home

## Appendix 5: Descriptive Statistics Key-Variables Related to Freedom, Government Quality, and Happiness

**Table:**

Variable	N	Min.	Max.	Mean	Std.
Global freedom 1–7	122	+1	+6.83	+3.1	1.77
Personal autonomy 1–16	120	+2	+16	+10.2	3.7
Press freedom 0–100	123	+10	+92	+46	21.4
Satisfaction with freedom 0–1	127	+.37	+.95	+.73	.14
Heritage Index 0–100	127	+23.2	+89.8	+62.0	10.1
Heritage Index Imp. ^a^0–100	127	+19.4	+90.3	+60.0	13.5
Fraser Index 0–10	127	+4.0	+9.0	+6.9	.78
Fraser Index Imp. ^a^0–10	127	+3.7	+8.9	+7.0	.98
Technical gov. quality −2,5 to + 2,5	127	−1.59	+2.06	+.09	.92
Happiness (life-ladder) 0–10	127	+2.94	+7.68	+5.48	1.11

^a^Improved; without components of size

## Appendix 6: Correlations Between Key-Variables Related to Freedom, Government Quality and Happiness

**Table:**

Variable	1	2	3	4	5	6	7	8	9
1. Global freedom	1								
2. Personal autonomy	.92	1							
3. Press freedom	.93	.87	1						
4. Freedom satisfaction	.33	.38	.34	1					
5. Heritage Index	.58	.65	.58	.45	1				
6. Heritage Index Imp.^a^	.68	.76	.69	.45	.96	1			
7. Fraser Index	.51	.57	.50	.41	.88	.84	1		
8. Fraser Index Imp.^a^	.59	.66	.59	.45	.90	.91	.95	1	
9. Technical gov. quality	.72	.80	.73	.55	.86	.94	.78	.88	1
10. Happiness (life-ladder)	.53	.60	.50	.58	.54	.61	.46	.55	.71

120–127 nations

All correlations are positive and significant (at .01 level)

^a^Improved; without components of size

## Footnotes

1 Christian Bay makes a similar distinction: between ‘psychological freedom’ on the one hand and ‘social’ and ‘potential freedom’ on the other. Psychological freedom is the degree of harmony between basic motives and overt behaviour. Social freedom is the relative absence of perceived external restraints on individual behaviour. Potential freedom is the absence of unperceived external restraints on individual behaviour (Bay 1958).

2 The distinction between ‘negative’ and ‘positive’ freedom is made, among others, by Fromm (1941), Berlin (1969) and Okulicz-Kozaryn (2014). There can be a tension between positive and negative freedom, as pointed out by Berlin. For example: if governments construct roads and bridges this will contribute to positive freedom, but it also has a negative impact on negative freedom if it leads to a higher level of taxation. In Berlin’s view negative freedom is the only ‘real’ freedom.

3 Veenhoven uses the phrase ‘political freedom’, but I will stick to the original phrase ‘global freedom’ as used by Freedom House. This is the average score for political rights and civil liberties. Just like Veenhoven I reverse scores to make sure that higher scores always indicate more freedom.

4 Spruk and Kešeljević (2015) also found a positive correlation between economic freedom and happiness.

5 There is discussion at this point. In his book ‘The Paradox of choice; Why More is Less’ (2004) Barry Schwartz argues that eliminating consumer choices can greatly reduce anxiety for shoppers. Paolo Verme (2009) argues that freedom can turn into a disutility if people expect they will be unable to handle freedom. Brulé and Veenhoven (2014) explain the difference in average happiness of the Finns and the French by a difference in the capability to choose, or the ‘psychological freedom’. But some incapability to choose is a specific problem, and Veenhoven can still be right that there are no ‘diminishing returns’ of negative or positive freedom, or the opportunity to choose as such, in the social or physical environment.

6 The World Bank measures six dimensions of the quality of government for all nations every year. The results are expressed in standardized scores, and the average of such scores is by definition 0. The averages in the sample of 127 nations are close to 0: −.05 for the democratic quality as the average of two dimensions, and +.09 for the technical quality as the average of four dimensions (on a range of −2,5 to + 2,5 in standard deviations). This is an indication that the representativeness of this sample is very acceptable. See “Appendix” for more information.

7 A Cronbach Alpha is often used as a reliability test to assess the reliability of a measurement to assess the consistency (correlations between items or questions). If the correlation between a specific indicator with the other indicators is low or negative, this indicator can be left out or the scale can be reversed. In this case I use it as a validity test to test the assumptions on which the measurement is based: do we measure what we expect to measure? Reversion of some item is therefore more than just a technical adaptation; it is also a reversion of some assumption.

8 Leaving these indicators out or reversing the signs also leads to a different outcome of a factor analysis: only one principal component instead of two components pointing in different directions. In the next section we get the same outcome for the measurement by the Fraser Institute, after leaving out ‘Size of Government’ or reversing the sign. These outcomes are better since there is no theoretical reason to expect more than one principal component for economic freedom.

9 The positive correlation between average happiness and ‘government consumption’ (+.50) and ‘transfers and subsidies’ (+,49) is remarkable, but government consumption and ‘transfers and subsidies’ refer to different government activities. More research is needed to explain the correlation with happiness. One hypothesis is that some types of ‘transfers and subsidies’ contribute to ‘decommodification’, as defined by Esping-Andersen (1990). See also Ott (2015).

10 The negative correlation between average happiness and ‘government enterprises and investments’ (−.41) is remarkable. One hypothesis for further research is that ex-communist nations still have high levels of government enterprises and investments and low levels of happiness. Another hypothesis might be that government involvement in the economy can easily become problematic, by unpredictable interventions in commercial management and ambiguities in responsibilities.

11 Respect for general principles of good governance is very important in contacts between citizens and government agencies. Well-known examples of such principles are: carefulness and accuracy in making decisions, correct communication of decisions, attention for all reasonable interests and expectations, no discrimination, no abuse of power, no disproportional negative consequences for citizens relative to public interests.

12 Researchers, like Bjornskov et al. (2007), and economists like Hayek (1944), are pessimistic. Researchers, like Persson and Rothstein (2015), and Radcliff (2013), are more optimistic. Some researchers are in favour of additional government activities because they are pessimistic about the effectiveness of free markets (North 1990; Lane 2000; Layard 2005).
